# Receding horizon control strategy for an electric vehicle with dual-motor coupling system in consideration of stochastic vehicle mass

**DOI:** 10.1371/journal.pone.0205212

**Published:** 2018-10-11

**Authors:** Hongqiang Guo, Jinyong Shangguan, Juan Tang, Qun Sun, Hongting Wu

**Affiliations:** 1 Department of School of Mechanical & Automotive Engineering, Liaocheng University, Liaocheng, Shandong Province, China; 2 Department of Zhongtong Bus Holding Co., Ltd, Liaocheng, Shandong Province, China; Beijing Institute of Technology, CHINA

## Abstract

Additional degrees of freedom existed in dual-motor coupling system bring considerable challenge to the optimal control of electric vehicles. Moreover, the stochastic characteristic of vehicle mass can further increase this challenge. A receding horizon control (RHC) strategy in consideration of stochastic vehicle mass is proposed in this study to respond to this challenge. Aiming at an electric vehicle with dual-motor coupling, a Markov chain is firstly deployed to predict future driving conditions by a formulated state transition probability matrix, based on historical driving cycles in real-world. Then, future required power is predicted by the predicted driving conditions, stochastic vehicle mass and road gradient, where the stochastic vehicle mass is formulated as stochastic variables in different bus stops. Finally, dynamic programming is employed to calculate the optimal vector of the vehicle within the defined prediction horizon, and only the first control values extracted from the optimal control vector are used to execute real-time power distribution control. The simulation results show that the proposed strategy is reasonable and can at least reduce electric consumption by 4.64%, compared with rule-based strategy.

## 1. Introduction

A sharp increase of vehicles is resulting in not only the depletion of oil energy but also the worsening of global environment [1]. Electric vehicles (EVs) characterized by zero-carbon emission and low energy consumption have attracted significant attention all over the world [2]. However, EVs usually have short driving ranges and high cost, compared with conventional vehicles [3]. Therefore, it is of great importance to investigate economical control strategy for EVs [4].

Different configurations of EVs have been proposed and investigated by researchers and automakers presently. The single-motor drive configuration is the most applicable solution for EVs, due to its simple structure and corresponding control strategy [5]. However, the motor power should be large enough to meet the acceleration and climbing requirements of EVs, which may greatly increase design cost and reduce economic performance [6–7]. To improve the dynamic and economic performances, motor equipped with automated manual transmission (AMT) configuration has been developed [8–9]. Owing to the advantages of large torque in low speed and large power in high speed of motor, AMT with two-speed ratio may be a convenient choice for EVs [10]. Moreover, because AMT with two-speed ratio has simplified shift actuator and control strategy, its development cost can be greatly reduced and its robustness can be significant improved [11]. Nevertheless, dynamic interruption is usually inevitable during shifting process. In contrast, dual-motor coupling system can avoid this problem if a well-designed coupler is equipped. For instance, an EV with a dual-motor coupling system has been proposed in Ref. [12–13]. To avoid the dynamic interruption problem, four driving modes including two four-wheel driving modes with low and high-speed ratios, front-wheel driving and rear-wheel driving modes with jointed torques are designed.

Since additional degrees of freedom lie in EVs with dual-motor coupling system, real-time control with high efficiency and good robustness is of great importance to EVs. In terms of this, many valuable strategies have been investigated, which can be roughly divided into four categories [14–15]. The first is rule-based control strategy. In Ref. [16], a rule-based control strategy with an average power distribution method is proposed. Fuzzy logic-based control strategies are also proposed to further improve the control performances [17–18]. However, although real-time control performance can be realized, the optimality is difficult to be guaranteed, due to the complexity of stochastic driving conditions. The second is intelligent control-based strategy. In Ref. [19], a neural network-based control strategy together with a hardware-in-loop experiment is proposed. For this, although real-time control performance and optimality can be ensured, large sampling sets should be acquired in a prior to train the neural network, which is usually difficult to be realized in real-world. The third is intelligent optimization-based control strategy. In Ref. [20], a genetic algorithm (GA) is deployed to optimize the non-linear power distribution for an EV. Theoretically, it is an efficient method to realize optimal control. However, real-time control performance may be dramatically sacrificed due to the iteration characteristic of GA. The forth is optimal control-based strategy such as dynamic programming (DP) and Pontryagin’s minimum principle (PMP) et al. As one of the most representative global optimization algorithms, DP has been widely deployed to find out the optimal shift schedule and power distribution for EVs [21–22]. Similarly, PMP is also employed to find out the optimal power distribution, by minimizing a designed Hamiltonian function [23–24]. Both of DP and PMP are promising methods for the optimal power distribution control. However, the prerequisite of known driving conditions usually restricts their application in real-world [25]. Besides, although many advanced power distribution strategies have been proposed, the factor of stochastic vehicle mass is seldom considered in the optimal control. This may greatly affect the optimality of the control strategies, especially for EVs with coupling system. Therefore, how to realize optimally real-time control in real-world considering stochastic vehicle mass is one of the most challenging issues for EVs with coupling system. In addition, if further driving conditions can be predicted, sub-optimal control can be realized by combining DP or PMP algorithm. Therefore, receding horizon control algorithm (RHC) constituted by driving condition prediction and DP may be a good choice for the economic improvement of EVs with coupling system [26].

The purpose of this paper is to address above issues by a proposed RHC strategy for an EV with dual-motor coupling system. Compared with the existed literatures, the main contributions of this paper are as follows:

1. A Markov chain is constructed for the RHC strategy based on historical driving conditions. It can predict future driving conditions such as acceleration and velocity trajectories, by a formulated state transition probability matrix;
2. The factors of stochastic vehicle mass and road gradient are considered in the prediction of the future required power, based on predicted driving conditions. Specially, stochastic vehicle mass is formulated as stochastic variables for bus stops;
3. DP is employed to calculate the optimal control vector (constituted by power distribution and shift instruction) for each receding horizon. Here, the optimal control values are the first control values extracted from the optimal control vector.

The remainder of this paper is organized as follows. Section 2 details the EV, including configuration, motor and battery models. Section 3 describes the RHC-based control strategy including Markov chain, DP and controlled models. The results and discussion of the control strategy are illustrated in Section 4, and conclusions are given in Section 5.

## 2. Configuration and models

### 2.1. Configuration

**Fig 1:**
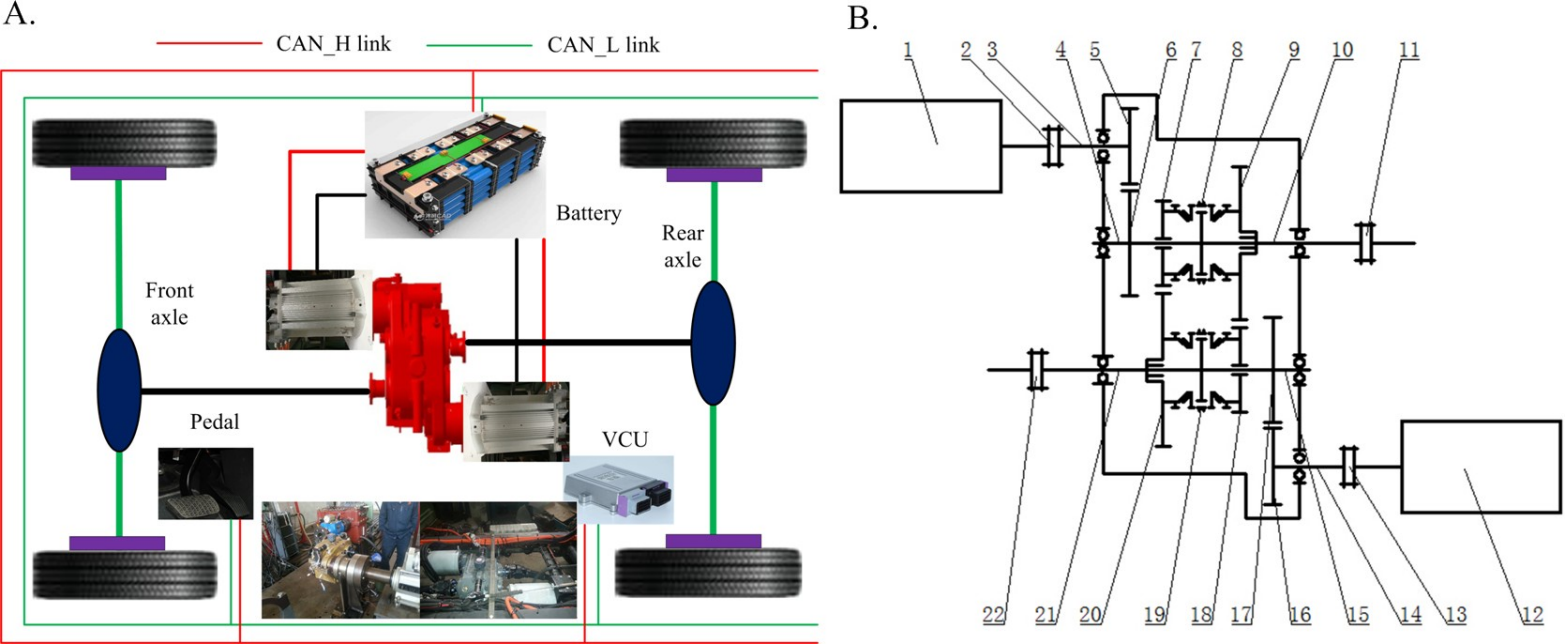
The configuration of the EV. **(A) The configuration of the vehicle; (B) The dual-motor coupling system.** In Fig 1A, the configuration of the vehicle is proposed and the vehicle communication is transmitted by controller area network (CAN) bus (CAN_H link and CAN_L link). In Fig 1B, the dual-motor coupling system is provided, and its parts are named as follows:1-The motor 1; 2, 11, 13, 22-The couplings; 3-The first input shaft; 5, 6, 7, 9, 16, 17, 18, 20-The constant meshing gears; 8, 19-The synchronizers; 10-The first output shaft; 12-The motor 2; 14-The second input shaft; 21-The second output shaft; 4, 15-The intermediate shafts; 23-The rear drive shaft; 24-The front drive shaft.

The configuration of the EV is shown in Fig 1A, which is characterized by a well-designed dual-motor coupling system. As shown in Fig 1B, the dual-motor coupling system is constituted by two motors and one coupler. The coupler has two input shafts, two countershafts, two output shafts, eight constant mesh gears and two synchronizers. Four driving modes including two single-axle driving modes and two four-wheel driving modes can be realized by controlling the two synchronizer sleeves. That is, if the synchronizers 8 and 19 simultaneously slide to left, joint forward driving mode can be realized; if the synchronizer 8 and 19 simultaneously slide to the right, joint backward driving mode can be realized; if the synchronizer 8 slides to right and the synchronizer 19 sliders to left, four-wheel driving mode with low speed ratio can be realized; if the synchronizer 8 slides to left and the synchronizer 19 sliders to right, four-wheel driving model with high speed ratio can be realized. In addition, it is worth noting that the speed ratios of the joint forward and backward driving modes are the same and are deployed as transition driving modes for the four-wheel driving modes. Besides, regenerative braking mode can be realized during deceleration and downhill.

The detailed parameters of the vehicle are shown in Table 1. For the coupler, the speed ratios constituted by the gears of 5 and 6, the gears of 16 and 17 are defined as primary gear ratio, denoted by *i*_g1_; the speed ratios constituted by the gears of 7 and 20, the gears of 9 and 18 are defined as secondary gear ratio, denoted by *i*_g2_. The output torques of the coupling system in different driving modes can be described as

$$\left\{ \begin{array}{l} T_{out1}=T_{1}\cdot i_{g1}\cdot i_{g2}+T_{2}\cdot i_{g1}\\ T_{out2}=T_{1}\cdot i_{g1}+T_{2}\cdot i_{g1}\cdot i_{g2}\\ T_{out3}=(T_{1}+T_{2})\cdot i_{g1}\\ T_{out4}=(T_{1}+T_{2})\cdot i_{g1}\cdot i_{g2} \end{array}\right.$$ (1)

where *T*_out1_ is the output torque of the joint forward driving mode; *T*_out2_ is the output torque of the joint backward driving mode; *T*_out3_ is the output torque of the four-wheel driving mode with low speed ratio; *T*_out4_ is the output torque of the four-wheel driving mode with high speed ratio; *T*_1_ and *T*_2_ denote the output torques of the motors, respectively; *i*_g1_ and *i*_g2_ denote the primary and secondary speed ratios, respectively.

**Table 1:** The parameters of the vehicle.

Item	Parameter	Value
**Vehicle**	Curb mass *m*	3000kg
	Passengers	10–18
	Tire radius *R*_*tire*_	0.473m
	Frontal area *A*_*area*_	4.3616 m^2^
	Rolling resistance coefficient *f*	0.012
	Air resistance coefficient *C*_*D*_	0.7
	Speed ratio of the main retarder *i*_*0*_	5.571
**Coupler**	Speed ratio *i*_g1_/*i*_g2_	1.26/2.12
**Motor**	Permanent magnet synchronous motor, max speed *n*_max_ /power *P*_max_	4000 rpm/70 kW
**Battery**	Nominal voltage/V	330V
	Capacity *Q*	72Ah

### 2.2. Motor model

**Fig 2:**
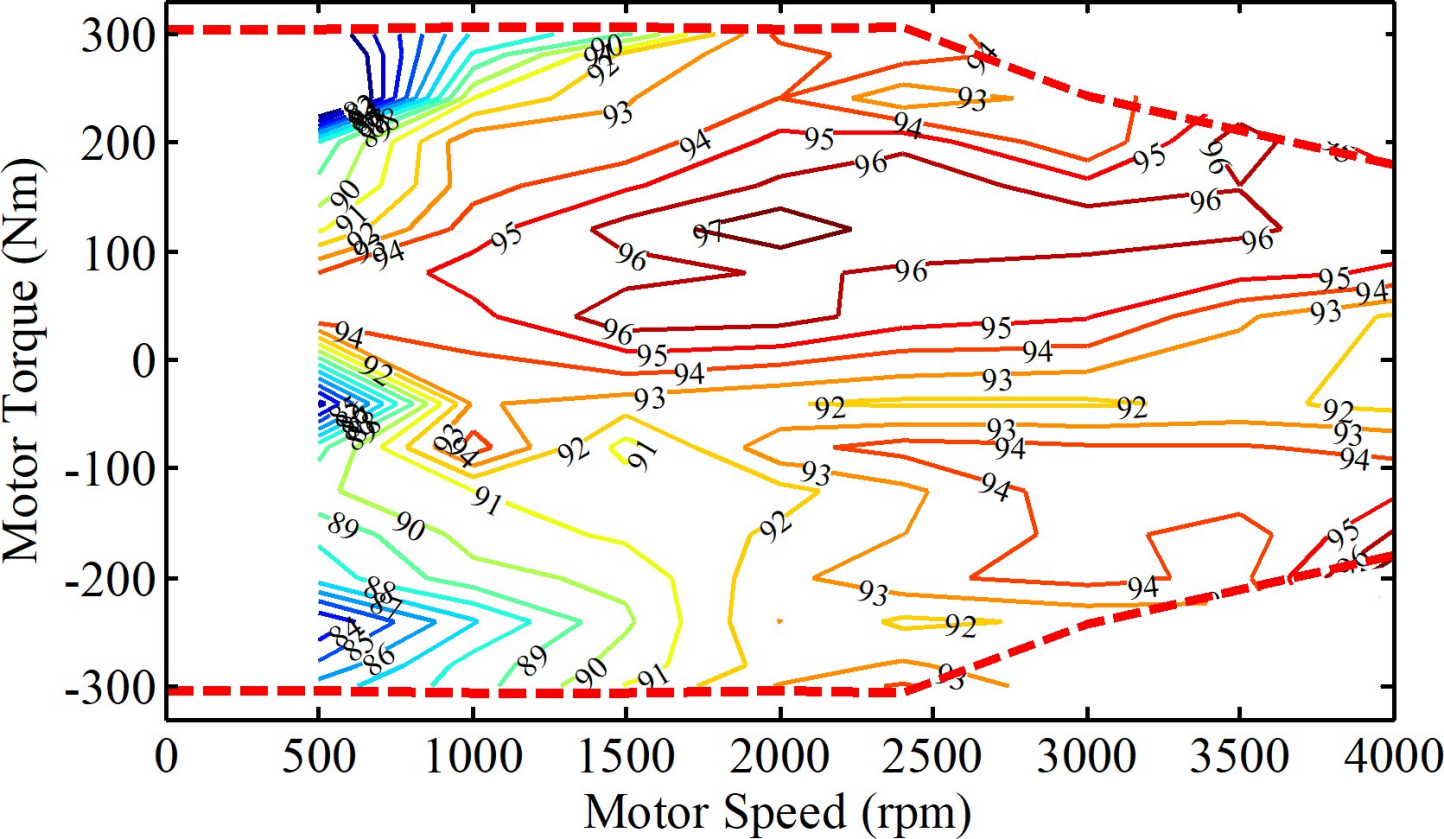
The efficiency MAP of the motor (%). This figure shows the motor efficiency with respect to different motor speeds and torques and the values of the efficiency is ranged from 80% - 97%.

Since the motors are the same, the speed of any motor can be described as

$$n=\frac{u}{0.377R_{tire}}i_{0}i_{g}$$ (2)

where *u* denotes the velocity of the vehicle; *R*_tire_ denotes the tire radius; *i*_0_ and *i*_*g*_ denote the speed ratios of the main retarder and the coupler, respectively. Since the motors can provide both driving torque and regenerative braking torque, the power of any motor can be presented by

$$P_{m}=\left\{ \begin{array}{l} \frac{T_{m}\cdot n}{9550}\cdot \eta_{m}\;\mathrm{if}\;P_{\mathrm{dem}}\geq 0\;(\mathrm{Driving}\;\mathrm{mode})\\ \frac{T_{m}\cdot n}{9550}\cdot \frac{1}{\eta_{m}}\;\mathrm{if}\;P_{\mathrm{dem}}\leq 0\;(\mathrm{Regenerative}\;\mathrm{braking}\;\mathrm{mode}) \end{array}\right.$$ (3)

where *T*_m_ denotes the torque of the motor; *η*_m_ denotes the efficiency of the motor, which can be obtained by interpolation method with the efficiency map of the motor in Fig 2; *P*_dem_ denotes the required power of the vehicle.

### 2.3. Battery model

**Fig 3:**
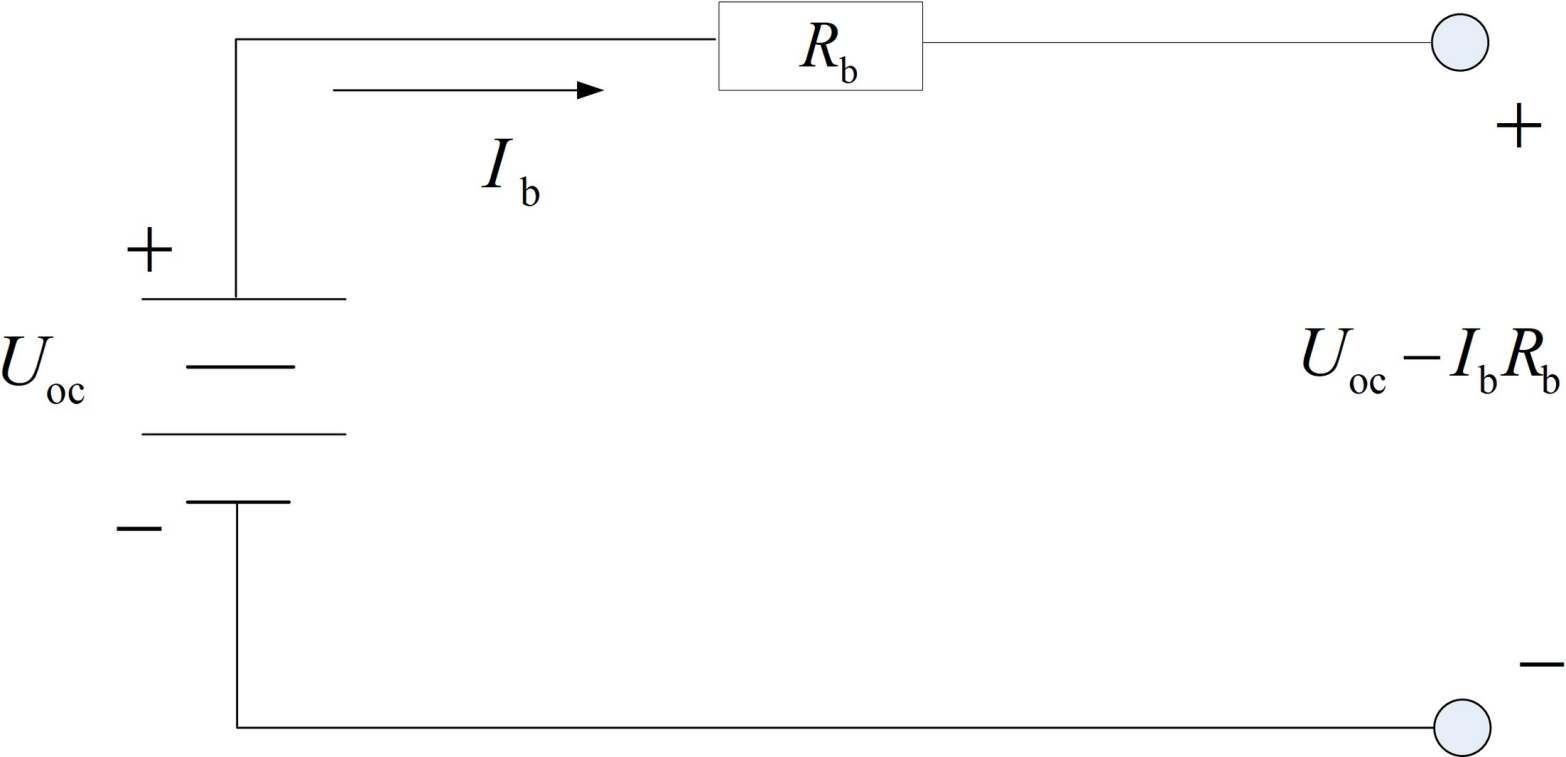
The Rint model of the battery. The figure shows the Rint model of the battery. The *P*_bat_ denotes the battery power; *I*_b_ denotes the battery current; *U*_oc_ denotes the open-circuit voltage; *R*_b_ denotes the internal resistance.

**Fig 4:**
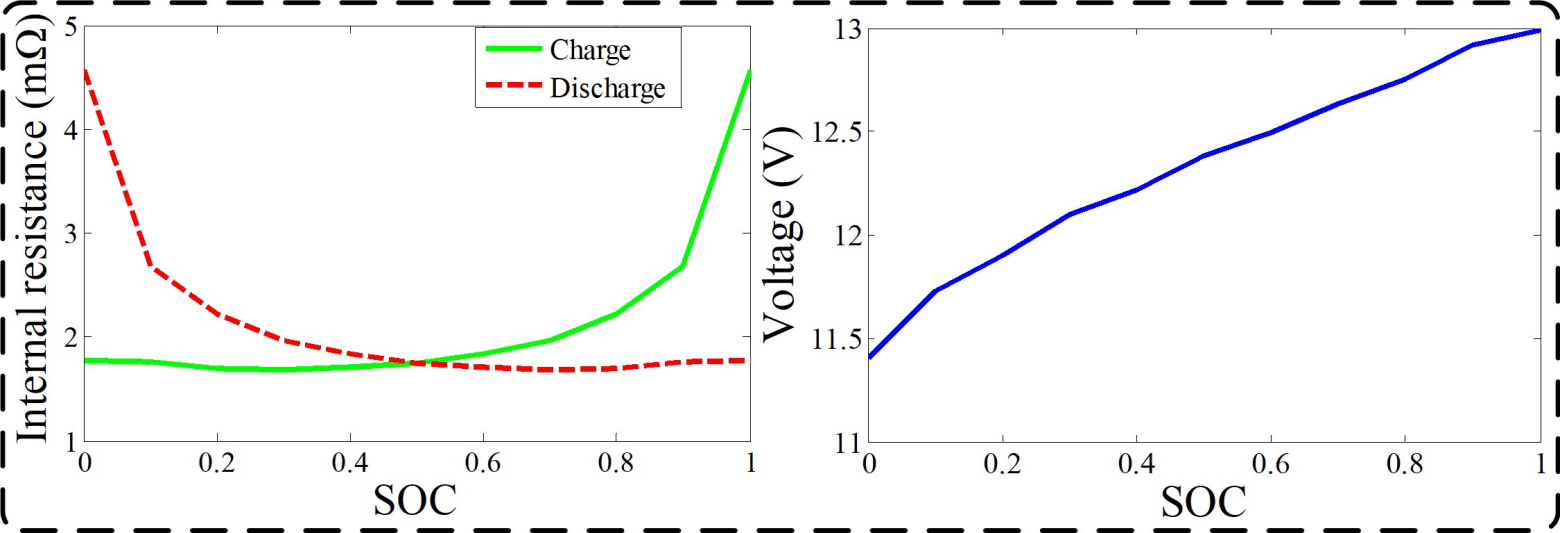
The internal resistance and open-circuit voltage of the battery. The figure shows the relationships among the internal resistance, the open-circuit voltage and state of charge (SOC).

As shown in Fig 3, a Rint model of the battery is employed to simplify the control problem. Based on the internal resistance and open-circuit voltage of the battery (Fig 4), the power and the current of the battery can be described as

$$\left\{ \begin{array}{l} P_{bat}=U_{\mathrm{oc}}I_{b}-I_{b}^{2}R_{b}\\ I_{b}=\frac{U_{\mathrm{oc}}-\sqrt{U_{\mathrm{oc}}^{2}-4R_{b}P_{\mathrm{bat}}}}{2R_{b}} \end{array}\right.$$ (4)

where *P*_bat_ denotes the battery power; *I*_b_ denotes the battery current; *U*_oc_ denotes the open-circuit voltage; *R*_b_ denotes the internal resistance.

## 3. RHC formulation

### 3.1. Framework

**Fig 5:**
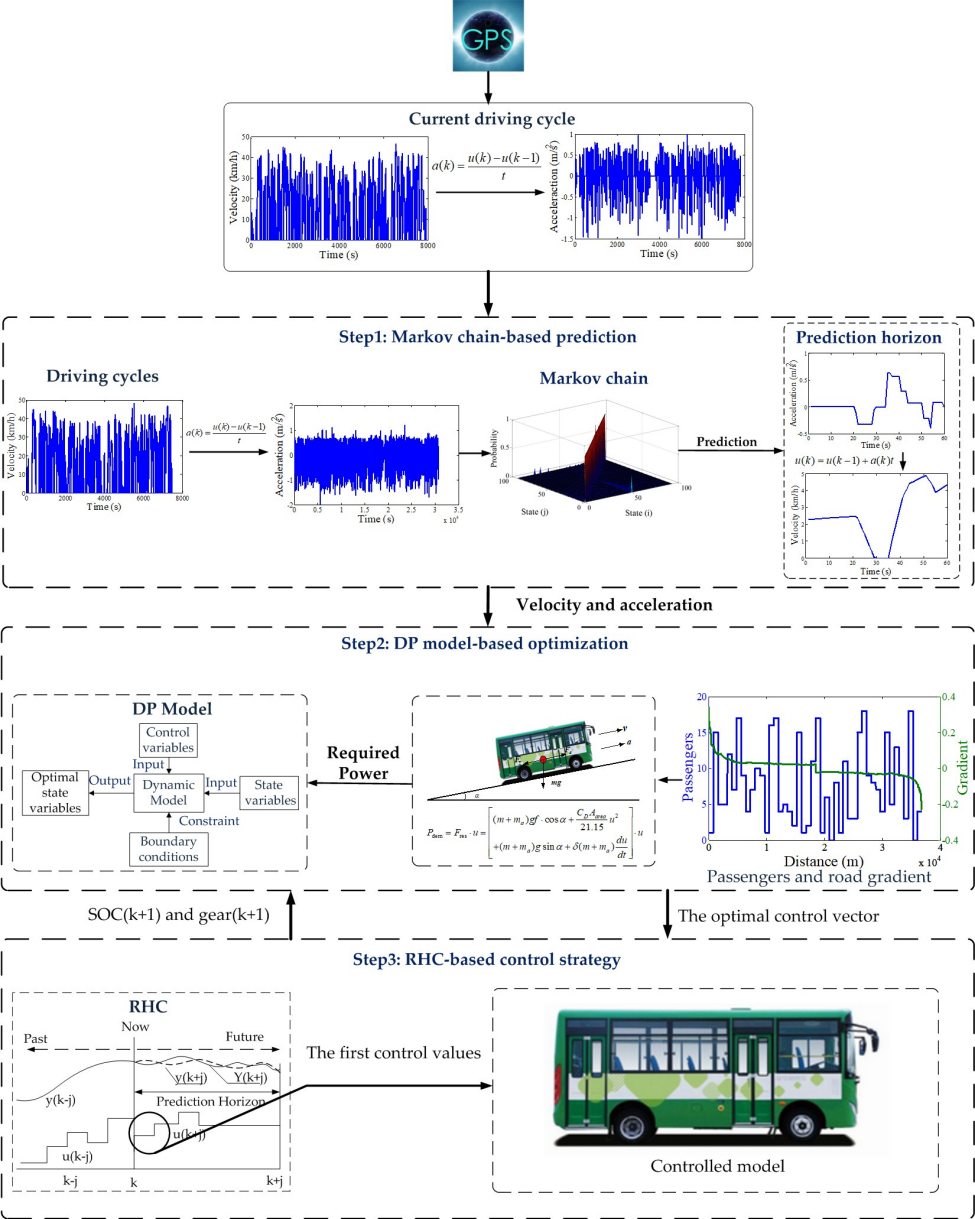
The Framework of the RHC strategy. The RHC strategy can be divided into three steps: Markov chain-based prediction, DP model-based optimization and RHC-based control strategy.

An RHC based strategy is designed in Fig 5, which is constituted by Markov chain-based prediction, DP model-based optimization and RHC-based control strategy steps.

Step1 (denoted by Markov chain-based prediction) is designed to predict acceleration and velocity trajectories within a defined receding horizon. A state transition probability matrix of acceleration is firstly formulated using a series of driving cycles in a fixed city bus route in real-world. Then, a Markov chain is built to predict future acceleration and velocity trajectories within the defined prediction horizon at every time step, based on the received information from GPS.

Step2 (denoted by DP model-based optimization) is designed to calculate the optimal power distribution and the optimal shift instruction based on DP. It is worth noting that two stochastic factors of vehicle mass and road gradient are considered in the model, due to their strong relationship with the required power of the vehicle.

Step3 (denoted by RHC-based control strategy) is designed to realize the real-time control of the vehicle. The first control values of the optimal control vector from DP are extracted to execute the power distribution of the two motors and the shift instruction of the coupler. Then, the updated SOC and gear position of the vehicle are taken as the initial state of the DP for the next time-step.

### 3.2. Markov chain-based prediction

#### 3.2.1 Driving cycles

**Fig 6:**
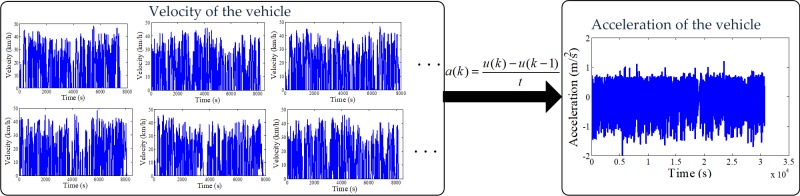
Historical driving cycles in real-world. The figure shows the historical velocities and accelerations from GPS.

One of the most key issues in the RHC strategy is the prediction of future driving conditions. Since historical driving cycles in real-world can be obtained from GPS, a Markov chain for future driving conditions prediction within a given prediction horizon can be constructed [27]. As shown in Fig 6, a series of driving cycles constituted by velocity are collected, and the corresponding acceleration sequence can be deduced by

$$a(k)=\frac{u(k)-u(k-1)}{t}$$ (5)

where *u*(*k*) and *u*(*k*−1) denote the current and previous velocities, respectively; *t* denotes the sampling time of the velocity.

#### 3.2.2. Markov chain

To predict future driving conditions, a state transition probability matrix should be firstly formulated. However, if it is formulated by velocity, only the number of states is discrete large enough, can prediction precision be satisfied. Therefore, a state transition probability matrix of acceleration is formulated in this paper.

**Fig 7:**
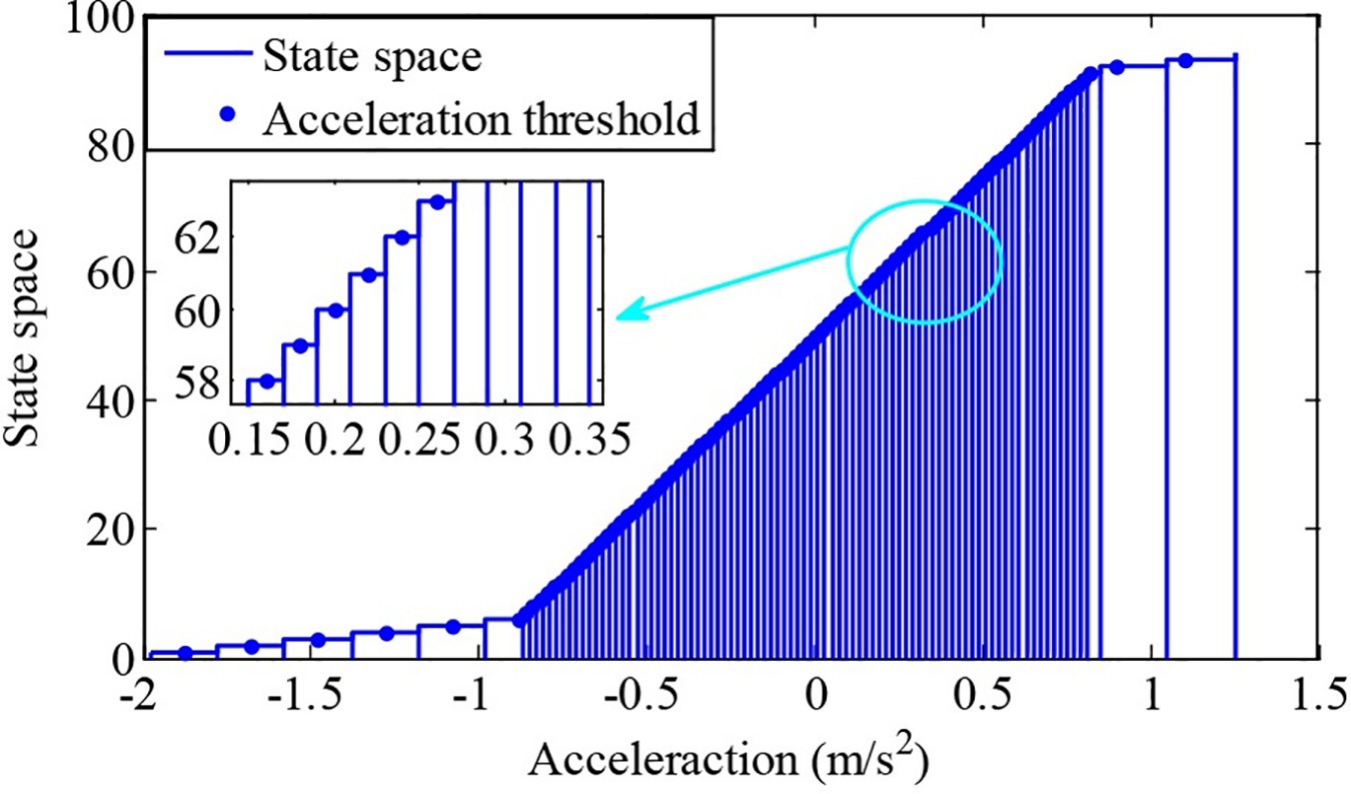
The state distribution of the acceleration. The figure shows the state distribution of the accelerations, where the state number is designed as 94 and the values are ranged from -2 to 1.5.

As shown in Fig 7, to ensure the predictive precision of future driving conditions, the state number of the acceleration is designed as 94 and the values are ranged from -2 to 1.5.

**Fig 8:**
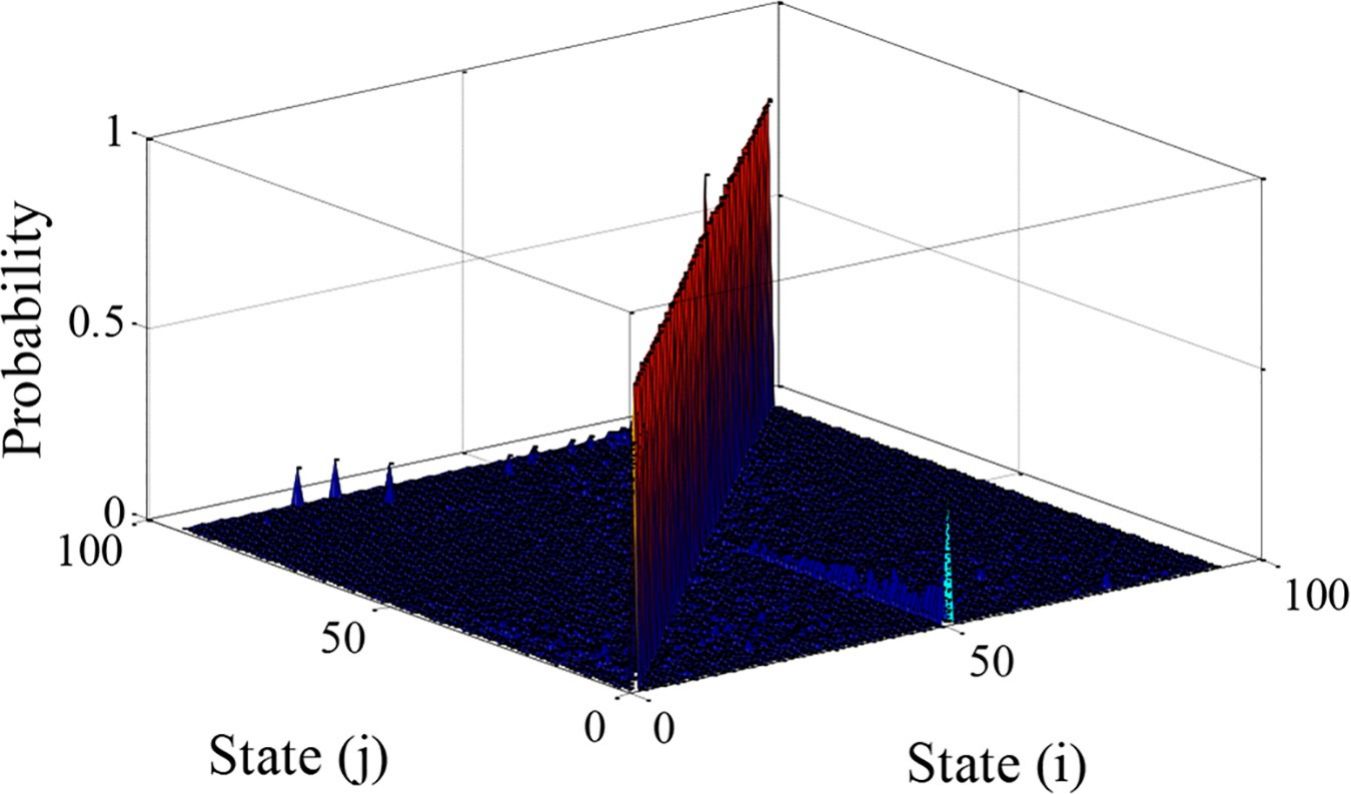
The state transition probability matrix of the acceleration. The state transition probability matrix is constructed by the sequence of accelerations in Fig 6.

Based on the theory of the Markov chain [28], the state transition probability matrix of the acceleration can be described as

$$P(a)=\left[\begin{array}{cccc} p_{11}(a) & p_{12}(a) & \cdots & p_{1n}(a)\\ p_{21}(a) & p_{22}(a) & \cdots & p_{2n}(a)\\ \vdots & \vdots & \ddots & \vdots \\ p_{n1}(a) & p_{n2}(a) & \cdots & p_{nn}(a) \end{array}\right]$$ (6)

where *n* denotes the number of the state; ${\hat{p}}_{i,j}(a)$ denotes the maximum likelihood estimation, which can be presented by

$${\hat{p}}_{i,j}(a)=\frac{{\hat{n}}_{i,j}(a)}{\sum_{j=1}^{n}{\hat{n}}_{i,j}(a)}$$ (7)

where ${\hat{n}}_{i,j}(a)$ denotes the number of the transfer from *i* to *j*, $\sum_{j=1}^{n}{\hat{n}}_{i,j}(a)$ denotes the total number of the transfer from *i* to *j* (*j* from 1 to *n*). The state transition probability matrix of the acceleration can be plotted by Fig 8.

### 3.3. DP model-based optimization

#### 3.3.1. Required power

As shown in Eq (8), the rolling resistance (*F*_f_), the road gradient resistance (*F*_i_) and the acceleration resistance (*F*_j_) have direct relationship with vehicle mass. This will further influence the optimal power distribution of the two motors and the optimal shift schedule of the coupler. Moreover, city buses usually show more stochastic characteristic than passenger cars with respect to the vehicle mass, due to the strong stochastic distribution characteristic of passengers in different bus stops. Therefore, the factor of the stochastic vehicle mass should be considered in power distribution control.

$$\left\{ \begin{array}{l} F_{f}=(m+m_{a})gf\cdot \cos\alpha \\ F_{i}=(m+m_{a})g\sin\alpha \\ F_{j}=\delta (m+m_{a})a \end{array}\right.$$ (8)

where *m* and *m*_*a*_ denote the curb mass and the passengers’ mass, respectively; *g* denotes the gravitational acceleration; *f* denotes the coefficient of rolling resistance; *α* denotes the angle of the road gradient; *δ* denotes the correction coefficient of rotating mass; *a* denotes the acceleration of the vehicle.

**Fig 9:**
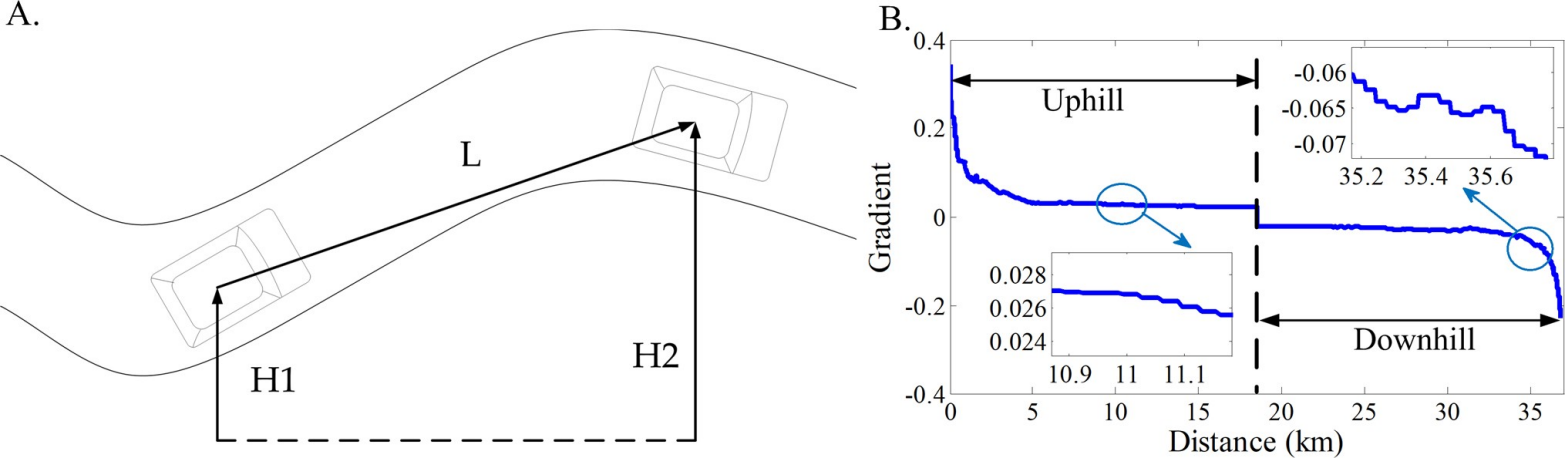
The road gradient of the fixed route. **(A) The sketch of the road; (B) The road gradient with respect to distance.** In Fig 9A, *H* is the road altitude and *L* is the trip distance. In Fig 9B, the uphill and downhill are included in the fixed route.

As shown in Fig 9A, road gradient can be estimated by trip distance and road altitude. Since their signals can be obtained from the GPS/GIS system, the road gradient can be deduced by

$$i=\tan(\arcsin\frac{H_{2}-H_{1}}{L})$$ (9)

where *H* denotes the road altitude; *L* denotes the trip distance. Since city bus usually has fixed route, the road gradient can be obtained off-line, based on the historical road information, and can be implemented into control strategy in a prior by taking the trip distance as an independent variable. As shown in Fig 9B, the road gradient of the fixed route changes sharply, where the negative values of the road gradient denote downhill, on the contrary, the meaning is just the opposite.

As stated above, the stochastic characteristic of vehicle mass and road gradient will greatly influence the optimal power distribution of the motors and the optimal shift schedule of the coupler. Therefore, the stochastic vehicle mass and road gradient should be considered in the required power prediction.

Based on the vehicle longitudinal dynamic equation, the traction of the vehicle can be described as

$$F_{\mathrm{res}}=(m+m_{a})gf\cdot \cos\alpha +\frac{C_{D}A_{area}}{21.15}u^{2}+(m+m_{a})g\sin\alpha +\delta (m+m_{a})a$$ (10)

where *F*_res_ denotes the traction of the vehicle; *C*_*D*_ denotes the coefficient of air resistance; *A*_*area*_ denotes the frontal area of the vehicle. Then, the required power of the vehicle can be presented by

$$P_{\mathrm{dem}}=F_{\mathrm{res}}\cdot u$$ (11)

where *P*_dem_ denotes the required power of the vehicle.

#### 3.3.2. DP model

Generally, the optimal control of the vehicle can be formulated as a discrete dynamic system, where DP can be deployed to solve it. In this paper, the state vector is designed as battery SOC, and gear position of the coupler. The control vector is designed as power distribution and shift instruction of the coupler. The discrete dynamic system can be described as

$$\left(\begin{array}{l} SOC(k+1)\\ g(k+1) \end{array}\right)=\left(\begin{array}{cc} 1 & 0\\ 0 & 1 \end{array}\right)\left(\begin{array}{l} SOC(k)\\ g(k) \end{array}\right)+\left(\begin{array}{c} \frac{1}{Q}I_{b}(k)\\ \beta_{s}(k) \end{array}\right)$$ (12)

where *SOC*(*k*) denotes the battery SOC; *g*(*k*) denotes the gear position; *β*_*s*_(*k*) denotes the shift instruction; *Q* denotes the rated battery capacity; *I*_b_ denotes the battery current. The battery is assumed to be fully charged at the beginning of the trip, which implies that the initial SOC is 0.8. *β*_*s*_(*k*) is designed as 1, 0 and -1 to denote upshift, hold on and downshift, respectively.

The optimization objective (Ψ(*k*)) is designed as minimizing the whole power consumption of the battery, which can be presented by

$$\Psi (k)=\left\{ \begin{array}{l} \left|\frac{P_{\mathrm{bat}}(k)}{P_{\mathrm{dem}}(k)}\right|\;\mathrm{if}\;P_{\mathrm{dem}}(k)\geq 0\;(\mathrm{Driving}\;\mathrm{mode})\\ \left|\frac{P_{\mathrm{dem}}(k)}{P_{\mathrm{bat}}(k)}\right|\;\mathrm{if}\;P_{\mathrm{dem}}(k)\leq 0\;(\mathrm{Regengerative}\;\mathrm{braking}\;\mathrm{mode}) \end{array}\right.$$ (13)

where *P*_dem_(*k*) denotes the required power of the vehicle.

Since the problem of frequent shifting of the coupler can greatly reduce the driving performance of the vehicle, it will be restricted in the cost-to-go function through the absolute of the shift instruction multiplied by a weighting factor (denoted by *γ*). Then, the cost-to go function of the EV can be described as

$$J_{\Phi }(x_{0})=\underset{N\to \infty }{\mathrm{Lim}}E\left\{\sum_{k=0}^{N-1}\left[L(\Psi (k))+\gamma \left|\beta_{s}(k)\right|\right]\right\}$$ (14)

where *J*_Φ_(*x*_0_) denotes the cost-to go function; *N* denotes the length of the receding horizon (60s). To ensure the reliable control of the vehicle, some physical constraints should also be defined as

$$\left\{ \begin{array}{l} P_{\mathrm{bat},\min}\leq P_{\mathrm{bat}}(k)\leq P_{\mathrm{bat},\max}\\ P_{m,\min}\leq P_{m}(k)\leq P_{m,\max}\\ n_{\min}\leq n(k)\leq n_{\max}\\ T_{m,\min}\leq T_{m}(k)\leq T_{m,\max}\\ 1\leq g(k)+\beta_{s}(k)\leq 3\\ SOC(k)-0.2\leq SOC(k)\leq SOC(k)+0.1 \end{array}\right.$$ (15)

where *P*_bat,min_ and *P*_bat,max_ denote the power boundaries of the battery; *P*_m,min_ and *P*_m,max_ denote the power boundaries of the motor; *n*_min_ and *n*_max_ denote the speed boundaries of the motor; *T*_m,min_ and *T*_m,max_ denote the torque boundaries of the motor.

### 3.4. RHC-based control strategy

#### 3.4.1. RHC principle

**Fig 10:**
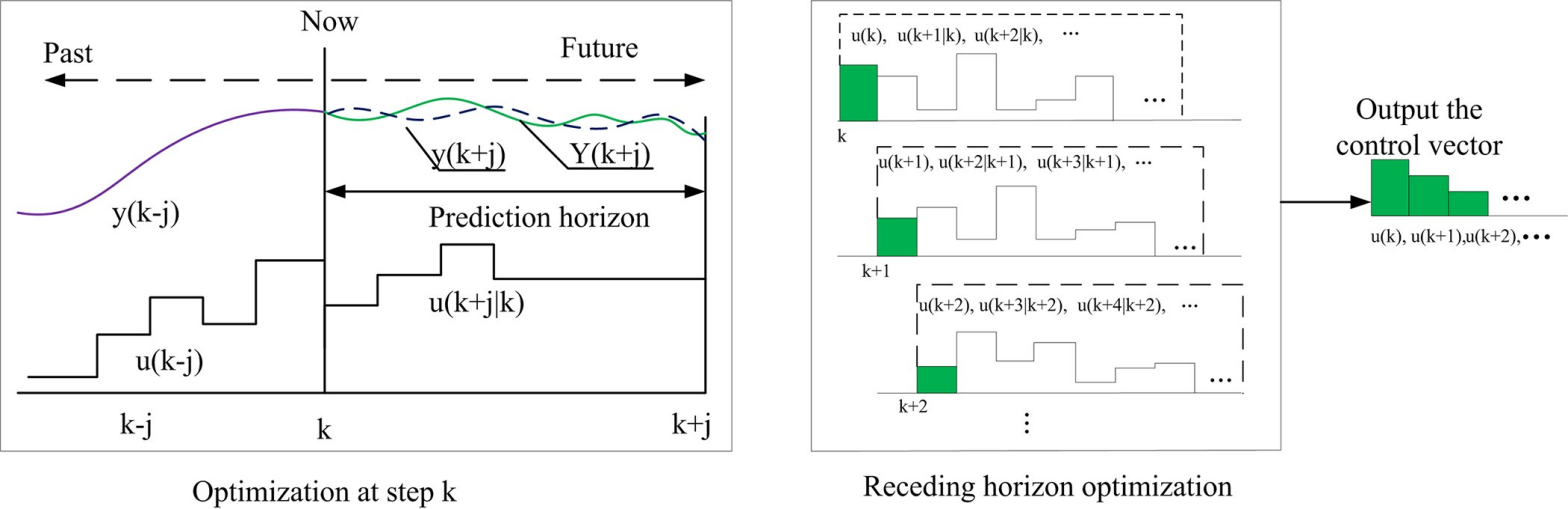
The RHC principle. The optimal control vector at time step of k will be obtained by DP, and the first one of the optimal control vector will be extracted to execute the power distribution control.

As shown in Fig 10, only the first control values extracted from the optimal control vector are applied to the controlled model. The initial states of the battery and coupler will be updated by the current output values.

#### 3.4.2. Controlled model

Based on the first control variables, the powers of the two motors can be described as

$$\left\{ \begin{array}{l} P_{m1}(k)=\chi (k)\cdot P_{dem}(k)\\ P_{m2}(k)=P_{dem}(k)-P_{m1}(k)\cdot \eta_{T}(k)\cdot \eta_{d}(k) \end{array}\right.$$ (16)

where *P*_m1_(*k*) and *P*_m2_(*k*) denote the powers of motors, respectively; *χ*^(*k*)^ denotes the optimal control value; *η*_*T*_(*k*) denotes the efficiency of the transmission shaft; *η*_m_(*k*) denotes the transmission efficiency of the drive axle.

Considering the efficiency of the motors, the power of the battery can be described as

$$P_{bat}(k)=\left\{ \begin{array}{l} \frac{1}{\eta_{1}}\cdot P_{m1}(k)+\frac{1}{\eta_{2}}\cdot P_{m2}(k)\;if\;P_{\mathrm{dem}}(k)\geq 0(Driving\;mode)\\ \eta_{1}\cdot P_{m1}(k)+\eta_{2}\cdot P_{m2}(k)\;if\;P_{\mathrm{dem}}(k)\leq 0(Regenerative\;braking\;mode) \end{array}\right.$$ (17)

## 4. Results and discussion

As state above, the stochastic vehicle mass in different bus stops has strong relationship with the optimal control of the vehicle. To exhaustively evaluate the robustness, application and optimality of the proposed RHC strategy, 46 stochastic variables with respect to bus stops are formulated to describe the stochastic distribution of the vehicle mass in different bus stops. Here, the stochastic vehicle mass is denoted by the mass of passengers, and assuming that the passenger’s mass is around 70kg. Besides, each stochastic variable is designed to 18 levels based on the maximum number of passengers. In this case, the stochastic variables of the bus stops will constitute a huge design space from the viewpoint of design of experiment. To better verify the proposed power distribution strategy, Opt LHD is employed to exhaustively insight into the design space.

As a representative method of design of experiment (DOE), Opt LHD has better performance than others to solve the spatial filling and equalization problems. It can better improve the sampling precision and robustness than other methods [29]. In specific, a designed *n*×*m* matrix *X* = [*X*_1_,*X*_2_,⋯*X*_*n*_]^*T*^ will be firstly generated by the stochastic LHD, where the vector of $X_{i}^{T}=[x_{i1},x_{i2},\cdots ,x_{im}]$ denotes experiment analysis and *m* denotes the number of bus stops. Then a new matrix will be regenerated by elemental exchanging. Finally, the optimal criteria of spatial filling can be achieved by the maximum distance criterion, which can be defined as

min1≤i,j≤n,i≠jd(Xi,Xj)=min[∑k=1m|Xik−Xjk|λ]1λ,λ=1or2 (18)

where *X*_i_ and *X*_j_ denote sampling points; *d*(*X*_i_,*X*_j_) denotes the distance between *X*_i_ and *X*_j_.

**Fig 11:**
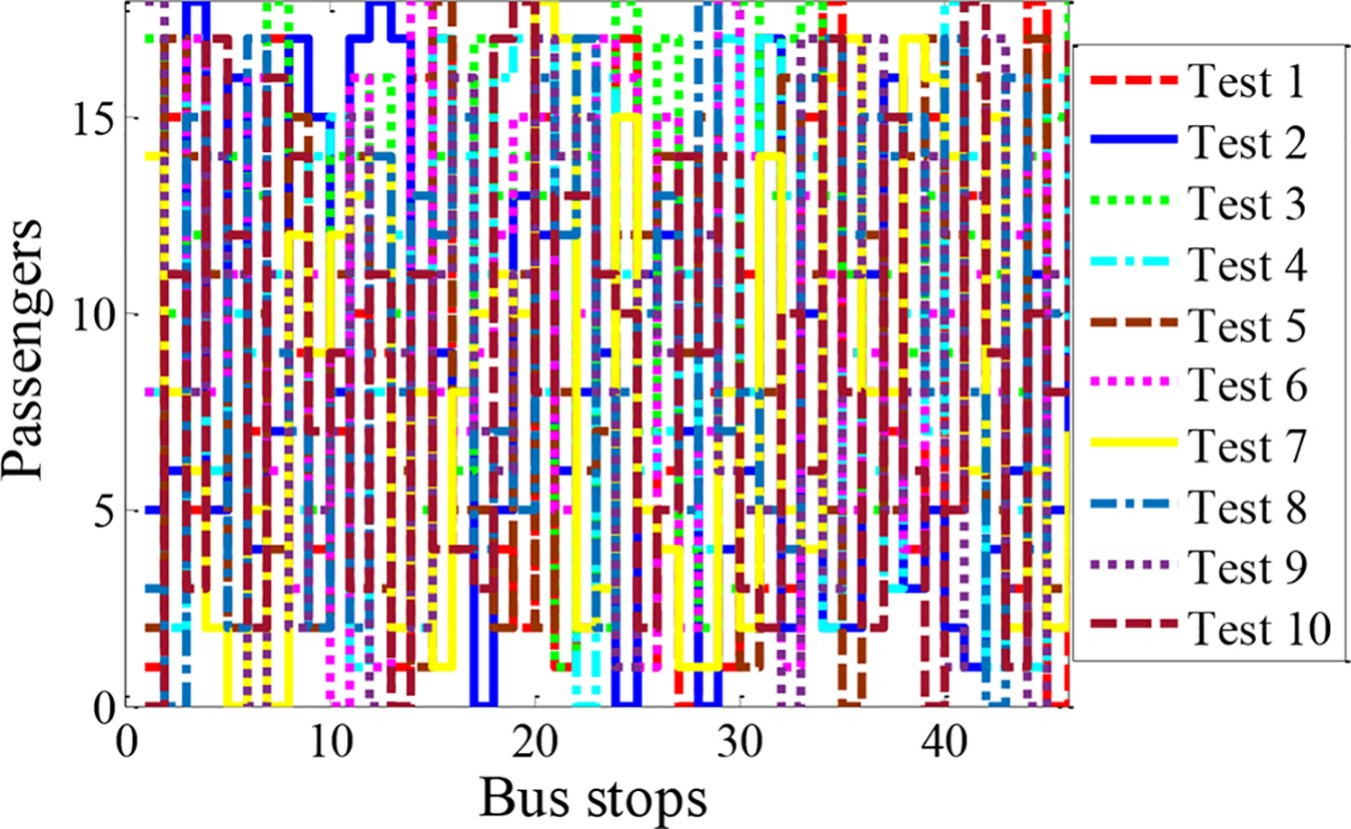
The stochastic distributions of passengers in bus stops. 10 tests are proceeded to verify the robustness and applicability of the proposed control strategy, based on Opt, LHD. The fixed route has 46 bus stops and the maximum passenger number is 18.

As shown in Fig 11, a series of stochastic distributions of passengers are presented to verify the robustness and application of the proposed control strategy, based on the sampling theory of the Opt, LHD. Here, the same colored line means the same sampling test, and 0 means that only driver on the bus.

In addition, ten compound driving cycles are constructed by three different driving cycles and the stochastic distributions of passengers (Fig 11), based on stochastic combination method. The trip times of the driving cycles are 7451s, 7811s and 8167s, respectively. The velocities are no more than 50km/h, and the distance of the whole trip is about 35km. The rule-based and DP control strategies are also employed to evaluate the proposed RHC strategy. The rule-based control strategy is defined as evenly power distribution of the two motors with a predefined shift schedule. DP is deployed to benchmark other strategies. Besides, the road gradient of the fixed city bus route has been implemented into the strategies in a prior.

**Fig 12:**
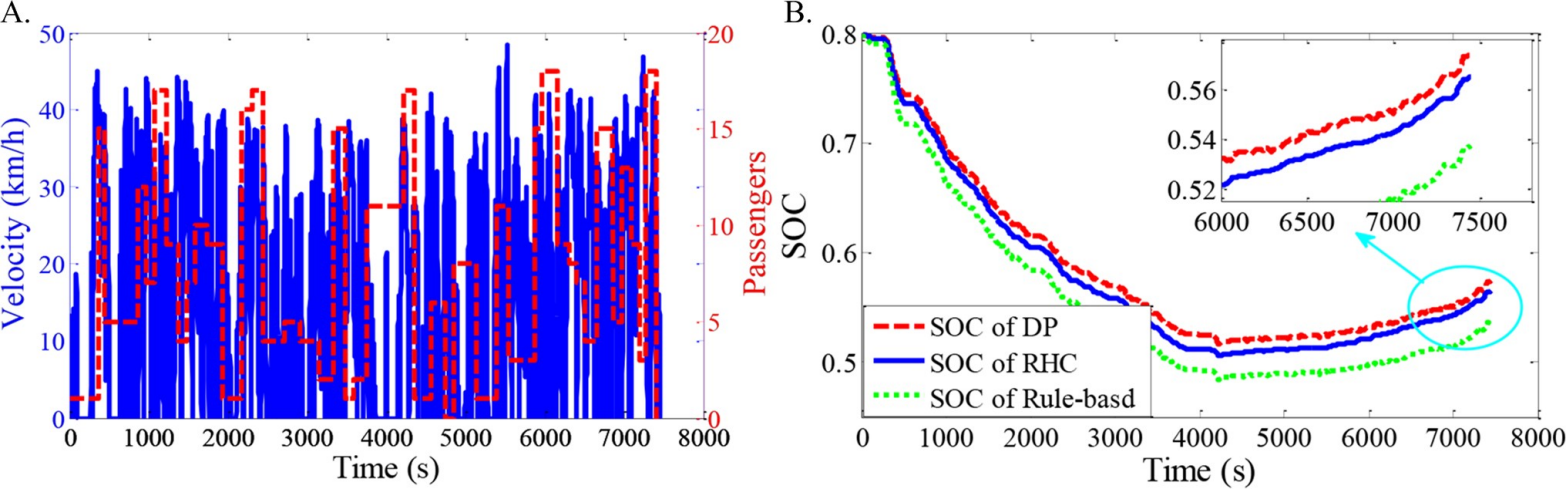
Simulation results with compound driving cycle 1. **(A) Compound driving cycle; (B) SOC trajectories of the strategies.** In Fig 12A, the velocity and the distribution of passengers in time domain are proposed. The total trip time is 7451s. In Fig 12B, the simulation results of DP, RHC and rule-based strategies are compared.

**Fig 13:**
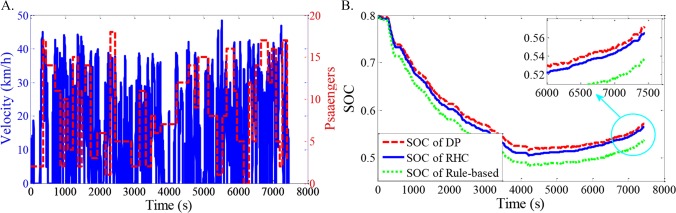
Simulation results with compound driving cycle 2. **(A) Compound driving cycle; (B) SOC trajectories of the strategies.** In Fig 13A, the velocity and the distribution of passengers in time domain are proposed. The total trip time is 7451s. In Fig 13B, the simulation results of DP, RHC and rule-based strategies are compared.

**Fig 14:**
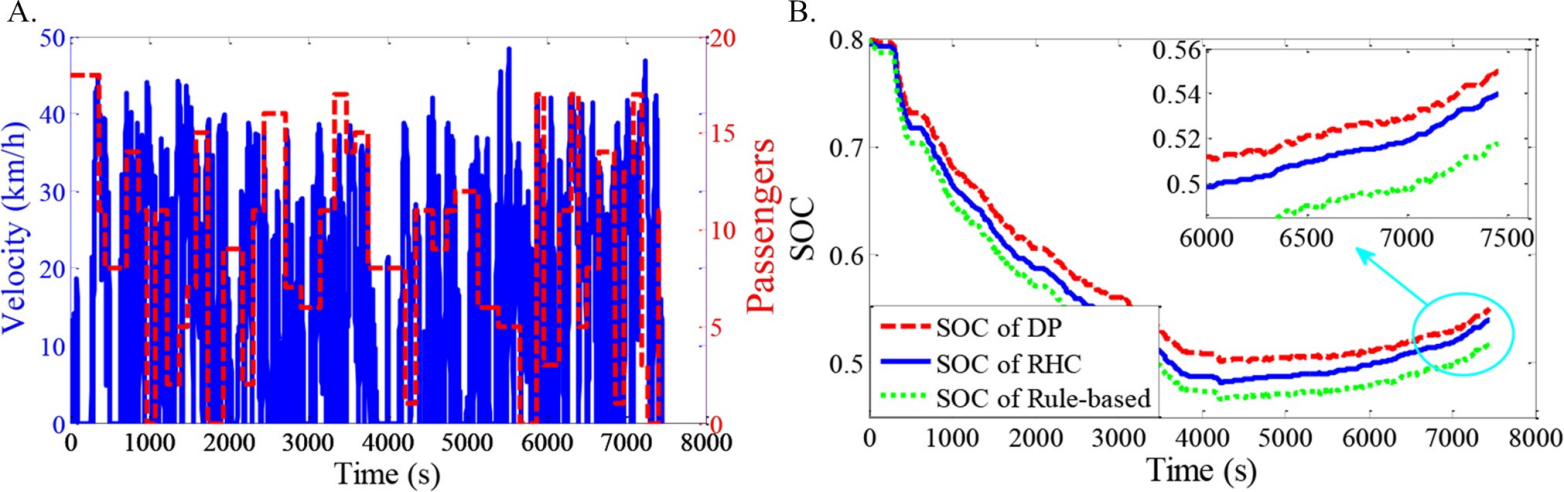
Simulation results with compound driving cycle 3. **(A) Compound driving cycle; (B) SOC trajectories of the strategies.** In Fig 14A, the velocity and the distribution of passengers in time domain are proposed. The total trip time is 7451s. In Fig 14B, the simulation results of DP, RHC and rule-based strategies are compared.

**Fig 15:**
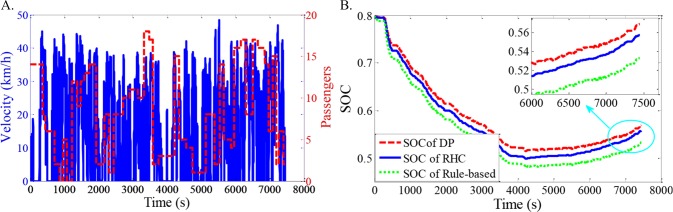
Simulation results with compound driving cycle 4. **(A) Compound driving cycle; (B) SOC trajectories of the strategies.** In Fig 15A, the velocity and the distribution of passengers in time domain are proposed. The total trip time is 7451s. In Fig 15B, the simulation results of DP, RHC and rule-based strategies are compared.

**Fig 16:**
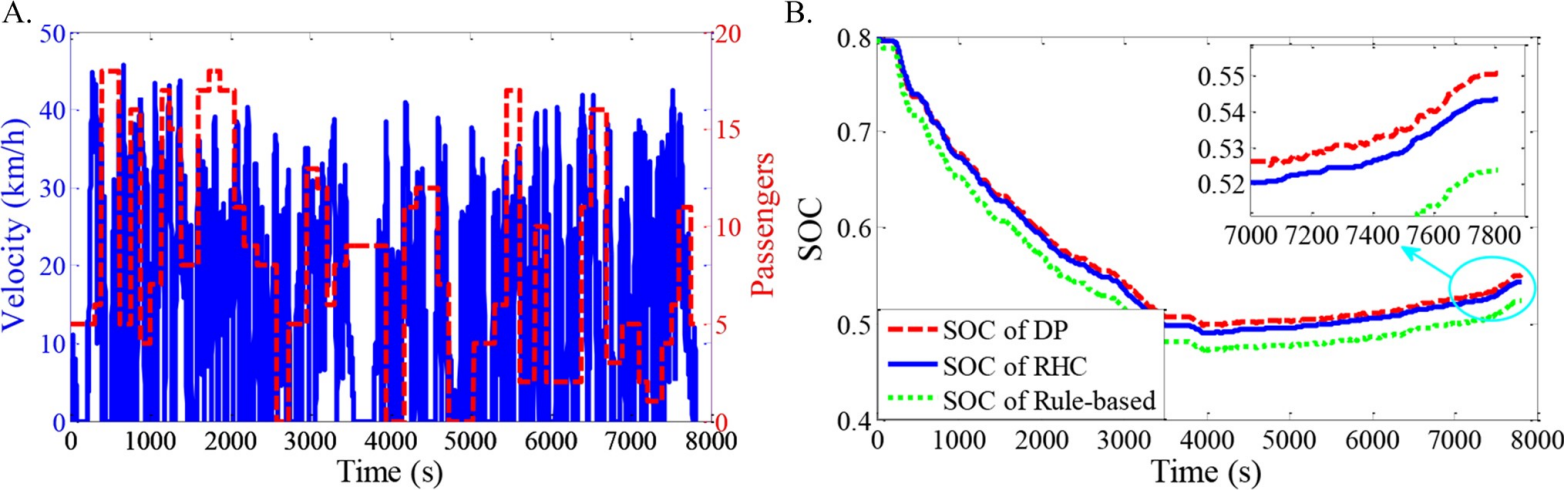
Simulation results with compound driving cycle 5. **(A) Compound driving cycle; (B) SOC trajectories of the strategies.** In Fig 16A, the velocity and the distribution of passengers in time domain are proposed. The total trip time is 7811s. In Fig 16B, the simulation results of DP, RHC and rule-based strategies are compared.

**Fig 17:**
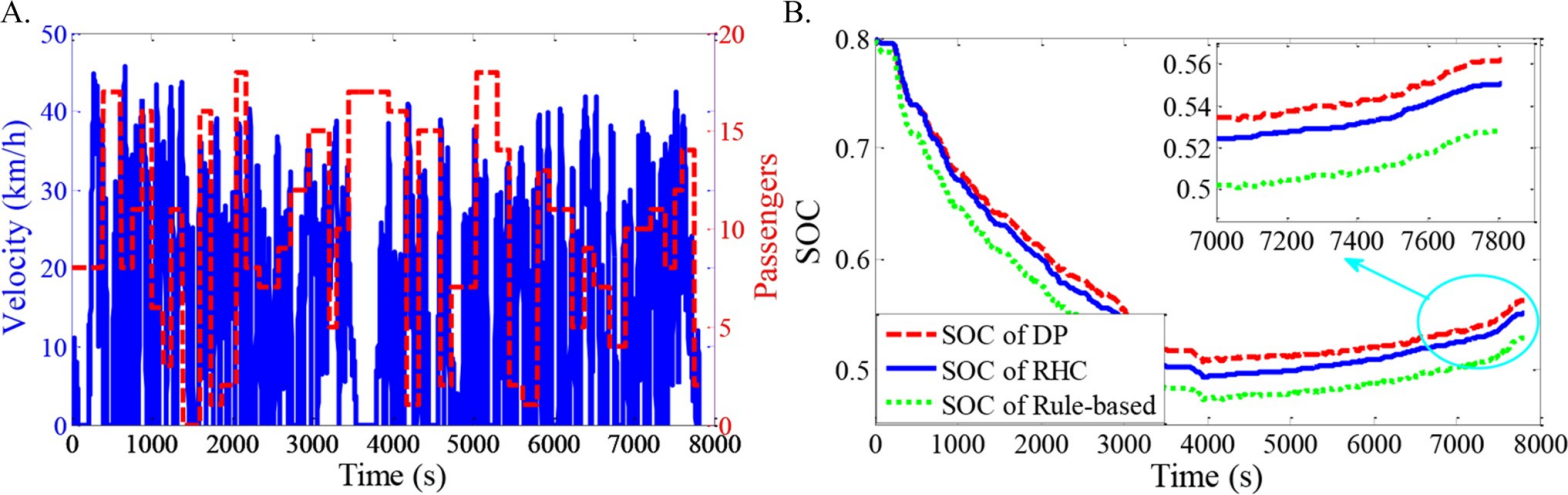
Simulation results with compound driving cycle 6. **(A) Compound driving cycle; (B) SOC trajectories of the strategies.** In Fig 17A, the velocity and the distribution of passengers in time domain are proposed. The total trip time is 7811s. In Fig 17B, the simulation results of DP, RHC and rule-based strategies are compared.

**Fig 18:**
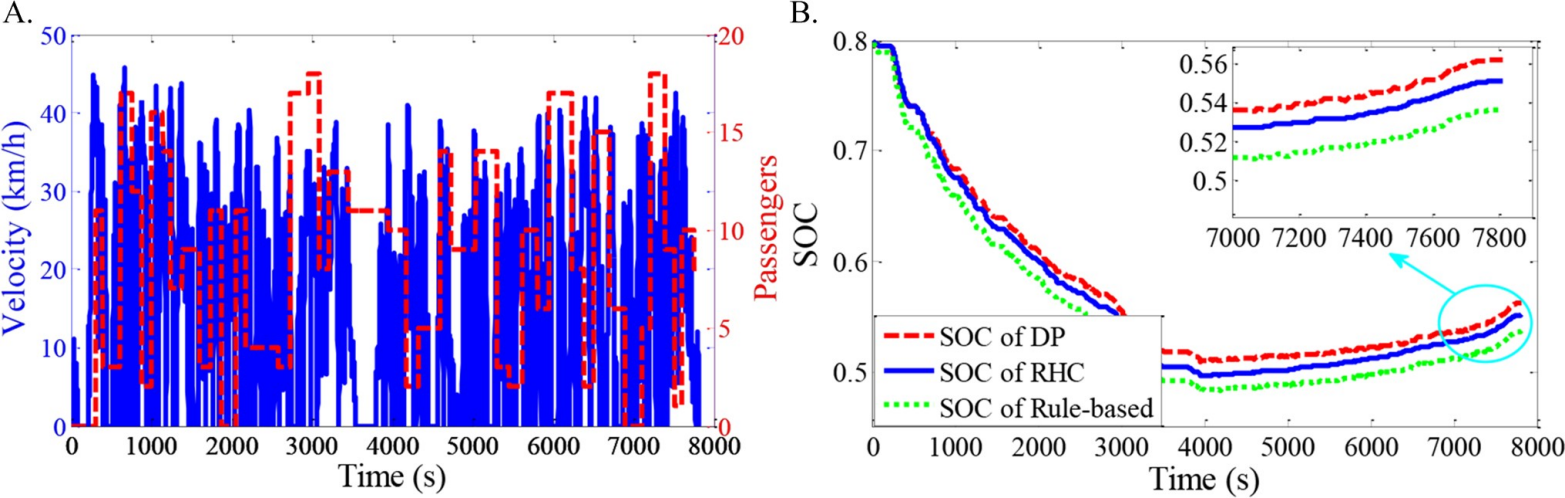
Simulation results with compound driving cycle 7. **(A) Compound driving cycle; (B) SOC trajectories of the strategies.** In Fig 18A, the velocity and the distribution of passengers in time domain are proposed. The total trip time is 7811s. In Fig 18B, the simulation results of DP, RHC and rule-based strategies are compared.

**Fig 19:**
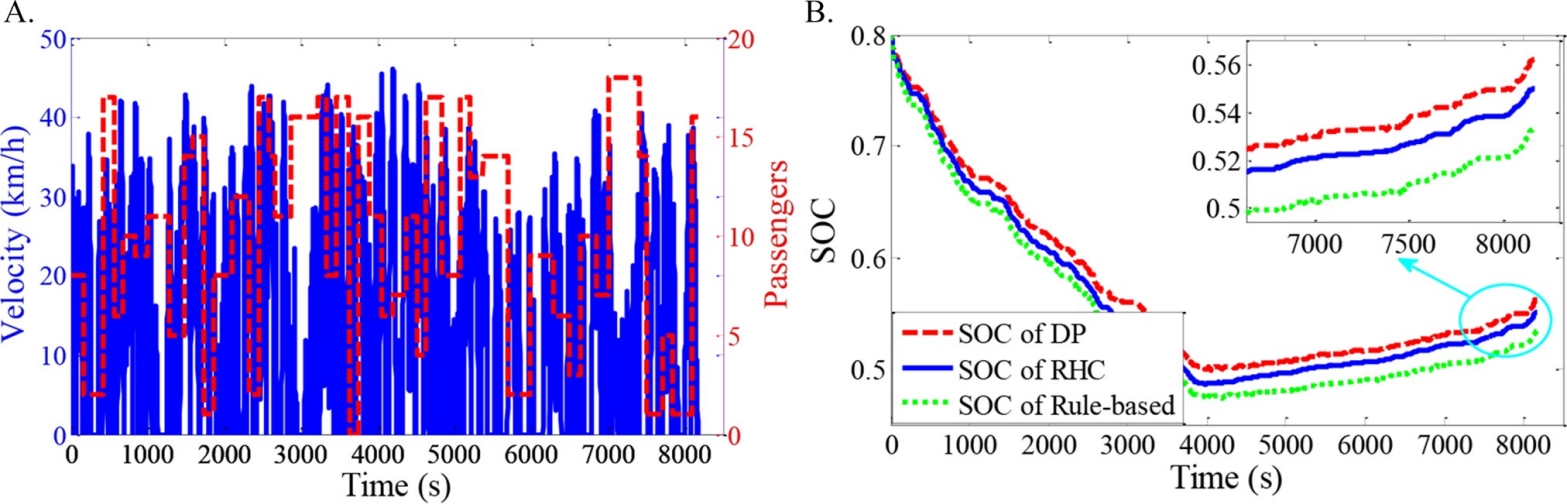
Simulation results with compound driving cycle 8. **(A) Compound driving cycle; (B) SOC trajectories of the strategies.** In Fig 19A, the velocity and the distribution of passengers in time domain are proposed. The total trip time is 8167s. In Fig 19B, the simulation results of DP, RHC and rule-based strategies are compared.

**Fig 20:**
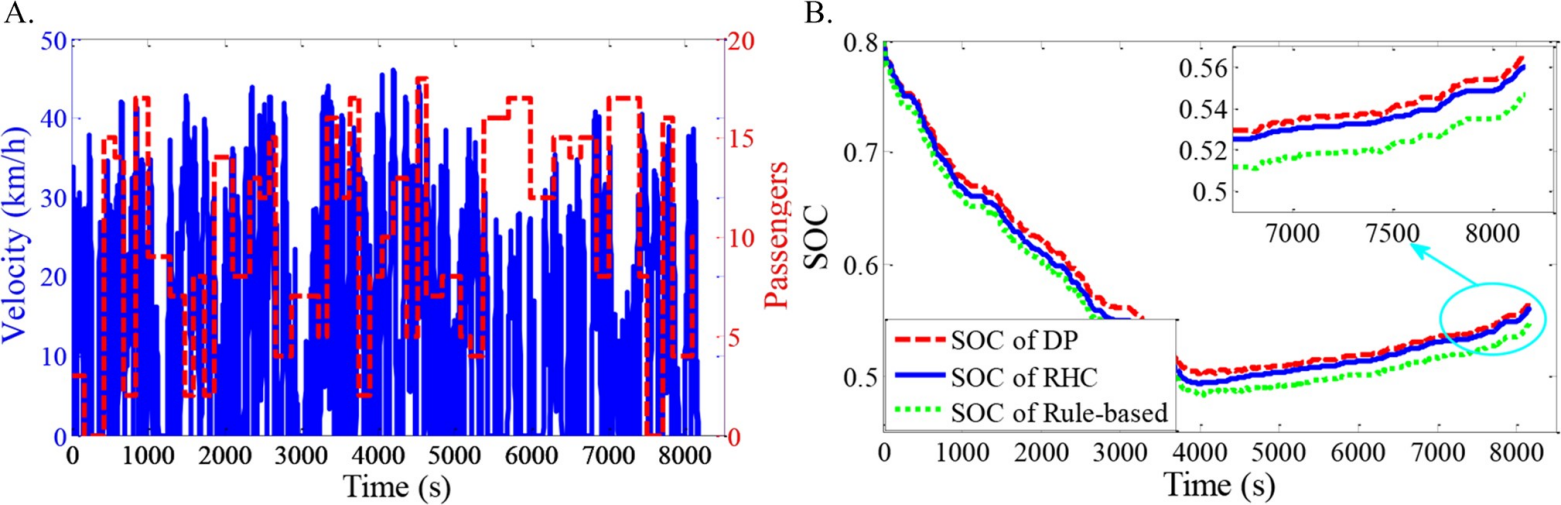
Simulation results with compound driving cycle 9. **(A) Compound driving cycle; (B) SOC trajectories of the strategies.** In Fig 20A, the velocity and the distribution of passengers in time domain are proposed. The total trip time is 8167s. In Fig 20B, the simulation results of DP, RHC and rule-based strategies are compared.

**Fig 21:**
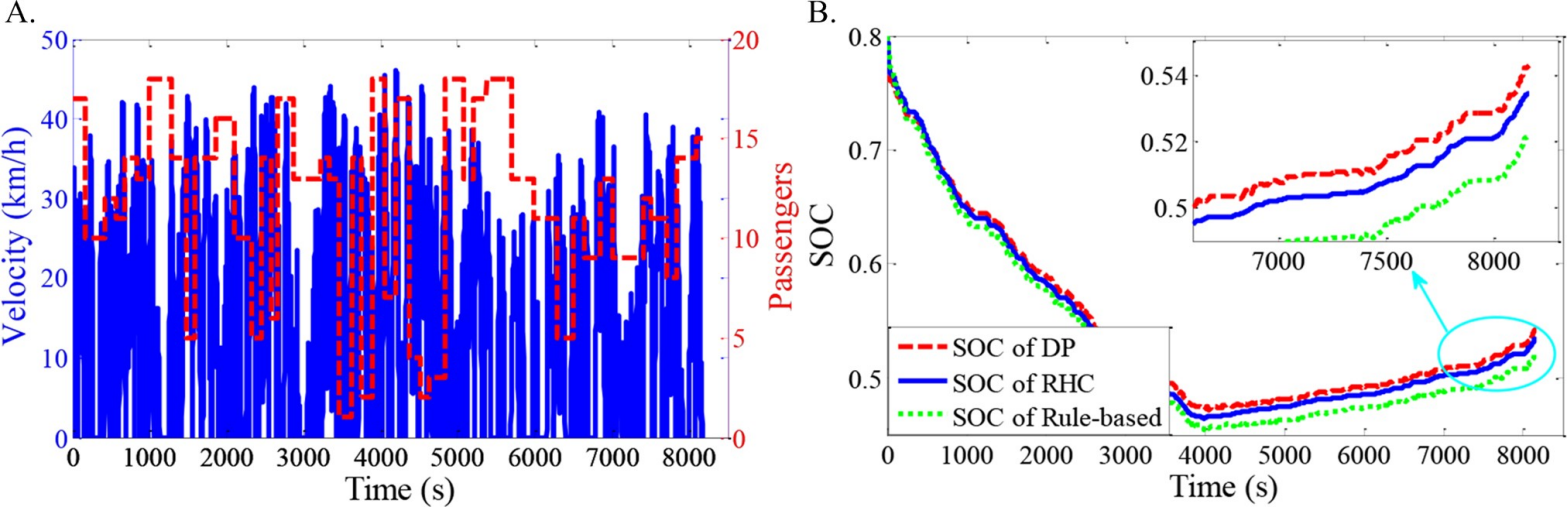
Simulation results with compound driving cycle 10. **(A) Compound driving cycle; (B) SOC trajectories of the strategies.** In Fig 21A, the velocity and the distribution of passengers in time domain are proposed. The total trip time is 8167s. In Fig 21B, the simulation results of DP, RHC and rule-based strategies are compared.

As shown in Figs 12–21B, all of the SOC trajectories of the RHC strategy are higher than the rule-based control strategy and lower than the DP control strategy. This implies that the proposed RHC strategy is better than the rule-based control strategy and worse than the DP control strategy. In addition, all of the SOC trajectories of the strategies decline sharply at the first half of the trip due to the larger required power on the uphill way, and they increase gradually at the second half of the trip, due to the frequent regenerative braking on the downhill way.

As shown in Table 2, all the terminal SOC values of the RHC control strategy are higher than the rule-based control strategy, and close to the DP control strategy. Since the control strategies have the same rated battery capacity and initial SOC, higher terminal SOC value implies lower electric consumption. Therefore, the RHC strategy has more energy saving potential than the rule-based control strategy and its economic performance approaches to the DP control strategy. Similarly, Table 3 also demonstrates the above conclusion. That is, the electric consumption of the RHC strategy can be at least improved by 4.64%.

**Table 2:** The terminal SOC values of the control strategies.

Compound driving cycles	DP strategy	RHC strategy	Rule-based strategy
**1**	0.5742	0.5648	0.5373
**2**	0.5715	0.5646	0.5367
**3**	0.5407	0.5396	0.5176
**4**	0.5686	0.5343	0.5214
**5**	0.5509	0.5437	0.5239
**6**	0.5618	0.5503	0.5279
**7**	0.5622	0.5512	0.5364
**8**	0.5622	0.5501	0.5331
**9**	0.5657	0.5598	0.5467
**10**	0.5427	0.5343	0.5214

**Table 3:** The electricity consumptions of the control strategies.

Compound driving cycles	RHC strategy (Ah)	Rule-based strategy (Ah)	Improvement
**1**	16.93	18.91	10.47%
**2**	16.95	18.96	10.60%
**3**	18.75	20.33	7.78%
**4**	19.13	20.06	4.64%
**5**	18.45	19.88	7.19%
**6**	17.98	19.59	8.22%
**7**	17.91	18.98	5.36%
**8**	17.99	19.22	6.40%
**9**	17.29	18.24	5.21%
**10**	19.13	20.06	4.64%

Furthermore, the electricity consumption (*Q*_c_) of the vehicle can be calculated by

$$Q_{c}=\left(SOC_{\mathrm{initial}}‑SOC_{\mathrm{final}}\right)\cdot Q$$ (19)

where *SOC*_initial_ denotes the initial SOC; *SOC*_final_ denotes the terminal SOC; *Q* denotes the rated battery capacity. The electricity consumptions of the strategies for different compound driving cycles are shown in Table 3.

## 5. Conclusions

This paper proposes an RHC strategy for an EV with dual-motor coupling system in consideration of stochastic vehicle mass. The conclusions are summarized as follows.

1. A Markov chain is constructed for the RHC strategy to predict acceleration and velocity trajectories, based on historical driving cycles in real-world. The stochastic vehicle mass is considered in the RHC strategy to improve its control performance.
2. To better evaluate the control performance of the RHC strategy, rule-based and DP control strategies are deployed. Moreover, Opt, LHD is employed to assist verifying the proposed control strategy, by exhaustively probing the design space (constituted by the designed stochastic variables).
3. The simulation results show that the electricity consumption of the RHC strategy closes to the DP control strategy and can be significantly reduced, compared with the rule-based control strategy.

The future work of this research will focus on the product-level application of the RHC strategy, and the implementation of state of health (SOH).
